# Treatment of Cutaneous Leishmaniasis With Cryosurgery and Itraconazole: A Case Report

**DOI:** 10.7759/cureus.89175

**Published:** 2025-07-31

**Authors:** Omar Gerardo Galaviz-Chaparro, María Fernanda Limón-Limón, Mariana Ceniceros-Cabrales, Víctor Manuel Tarango-Martinez

**Affiliations:** 1 Dermatology, Instituto Dermatológico de Jalisco "Dr. José Barba Rubio", Zapopan, MEX; 2 Dermatology, Instituto Dermatológico de Jalisco “Dr. José Barba Rubio", Zapopan, MEX

**Keywords:** cryosurgery, cutaneous leishmaniasis, itraconazole, leishmania mexicana, leishmaniasis

## Abstract

Cutaneous leishmaniasis is an endemic parasitic disease in parts of Latin America, caused by various *Leishmania* species. While pentavalent antimonials are considered first-line treatment, their toxicity and limited availability in some regions necessitate alternative therapeutic strategies. We present a 25-year-old immunocompetent male who developed four painless, erythematous-violaceous lesions one month after travel to Tulum, Mexico. The lesions measured between 0.8 and 2.5 cm and were located on the ear, axilla, and back. Direct smear and molecular techniques confirmed infection by *Leishmania mexicana*. The patient was treated with oral itraconazole (200 mg/day for three months) and two cryotherapy sessions performed four weeks apart. Complete clinical resolution was achieved and sustained at one-year follow-up. This case highlights the effectiveness of combining itraconazole and cryotherapy for cutaneous leishmaniasis due to *L. mexicana*, particularly in settings with limited access to standard therapies. Early diagnosis and individualized treatment approaches can optimize patient outcomes.

## Introduction

Leishmaniasis is a vector-borne infectious disease caused by protozoa of the genus *Leishmania*, with an estimated annual incidence of 1.5 to two million new cases worldwide [1,2]. The parasite is classified into Old World and New World species;* Leishmania mexicana* is endemic to the latter, particularly in regions such as Mexico and Central America. Clinically, leishmaniasis manifests in three major forms, namely, cutaneous, mucocutaneous, and visceral, with cutaneous leishmaniasis being the most frequently encountered.

According to current clinical practice guidelines [3], local therapy is preferred for uncomplicated cutaneous forms of Old World leishmaniasis. Treatment modalities include topical heat or cryotherapy, either alone or combined with agents such as paromomycin, intralesional antimonials, photodynamic therapy, or laser-based interventions [2,3]. However, there is limited evidence regarding the efficacy of combined systemic and local treatments for cutaneous leishmaniasis caused by New World species, particularly in contexts with restricted access to first-line therapies.

We present a case of cutaneous leishmaniasis caused by *L. mexicana*, successfully treated with a combination of oral itraconazole and cryotherapy. This report aims to contribute to the growing body of literature on alternative therapeutic strategies in resource-limited settings, where conventional treatments may be unavailable or contraindicated.

## Case presentation

**Figure 1:**
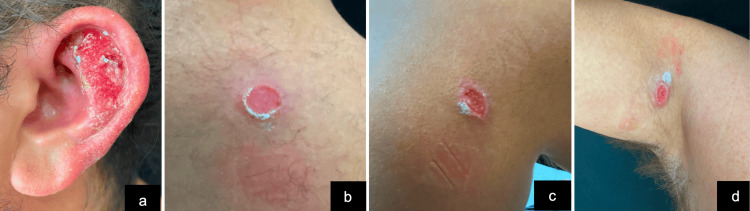
Initial clinical presentation (day 30 post-exposure). Erythematous-violaceous plaque and ulcers on the left ear, right axilla, and upper back.

A 25-year-old male presented 30 days after traveling to Tulum, Quintana Roo, Mexico, with a moist plaque featuring superficial crusts, irregular borders, measuring 2.5 x 2 cm, along with three ulcers ranging from 0.8 to 1.5 cm in diameter, which were erythematous to violaceous, with serohemorrhagic exudate and a fibrinoid appearance. These lesions were located on the left ear (involving the antihelix and the scaphoid fossa), the right axilla, and the back. They were painless with occasional pruritus (Figure 1). 

**Figure 2:**
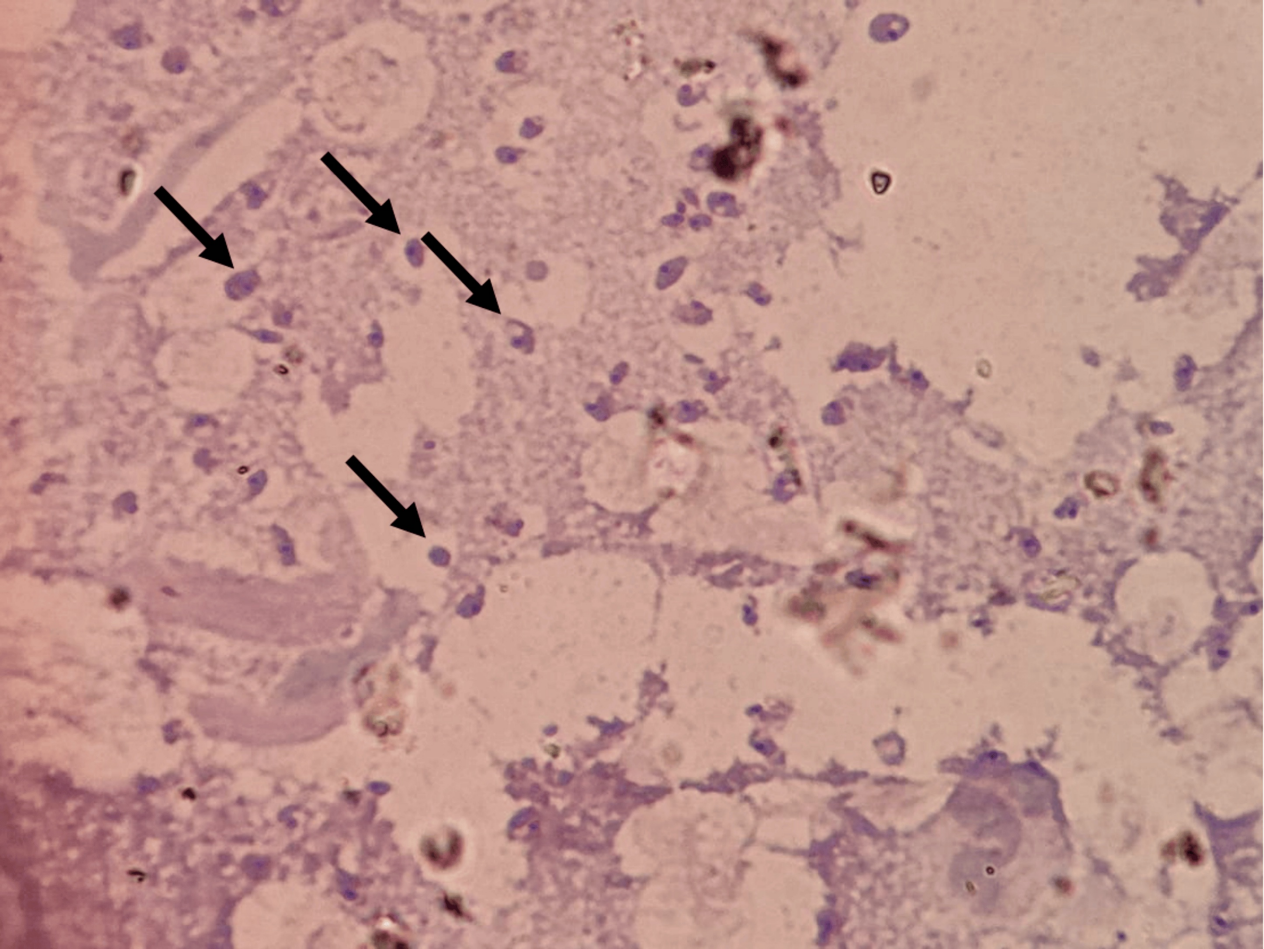
Giemsa-stained microphotograph showing abundant intracellular Leishmania amastigotes.

Laboratory tests, including HIV serology, were within normal limits. Wright and Giemsa stains revealed abundant extracellular amastigotes with a parasitic density of +10. Histopathological examination showed a dense inflammatory infiltrate composed of lymphocytes, histiocytes, and multinucleated giant cells (Figure 2). Molecular identification using polymerase chain reaction (PCR) combined with restriction fragment length polymorphism (RFLP) analysis confirmed the species as *Leishmania mexicana mexicana*.

**Figure 3:**
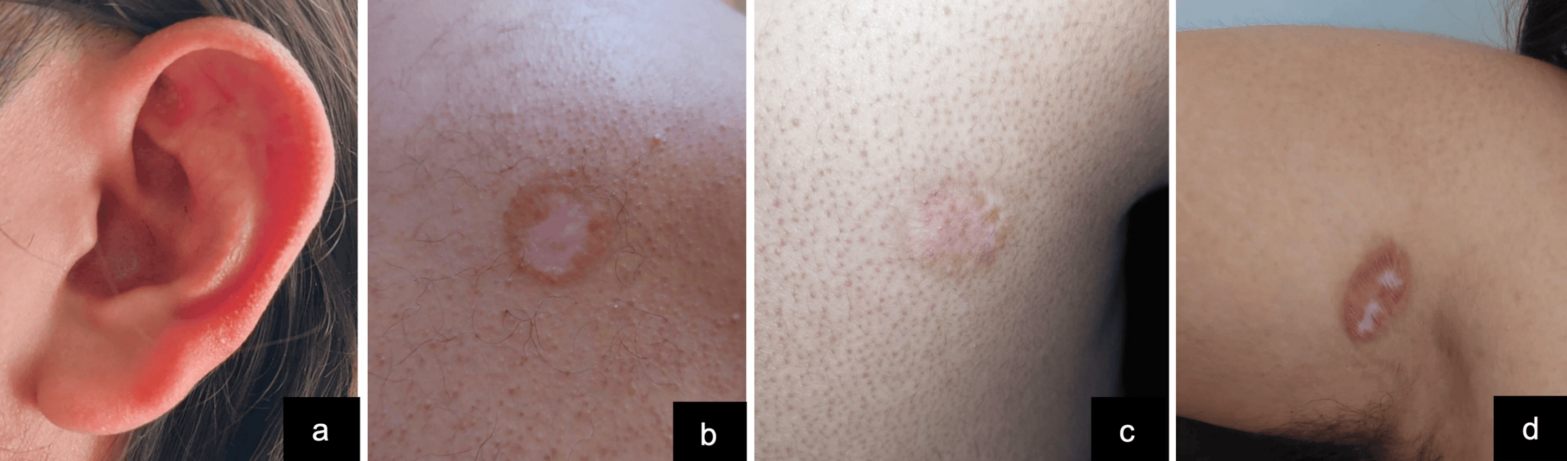
Clinical outcome (one year post-treatment). Complete reepithelialization and absence of active lesions.

Treatment included oral itraconazole 200 mg daily for three months and two sessions of cryotherapy performed four weeks apart. Each session consisted of two 45-second freeze cycles. At the one-year follow-up, no evidence of lesion recurrence was observed. The treated areas showed complete reepithelialization with minimal postinflammatory hyperpigmentation and no residual scarring or deformity. The patient reported no functional limitations, particularly in the auricular region (Figure 3).

## Discussion

Leishmaniasis is a chronic parasitic disease that manifests in three clinical forms: cutaneous, mucocutaneous, and visceral. In Mexico, the states with the highest number of cases are Tabasco and Quintana Roo [3]. The disease is caused by several protozoan species of the genus *Leishmania*, classified into Old World species (*L. major, L. infantum, *and *L. tropica*) and New World species (*L. amazonensis, L. chagasi, L. mexicana, L. naiffi, L. braziliensis, *and *L. guyanensis*).

There is no universally ideal therapy. Criteria for local treatment include the presence of fewer than four lesions, each measuring 5 cm or less, without regional lymphadenopathy, located in areas amenable to local therapy, in immunocompetent patients, with no history of treatment failure [2,3]. First-line treatment typically involves pentavalent antimonials, which are effective but may be associated with significant toxicity. Adverse effects include cardiotoxicity and nephrotoxicity [4]. Second-line treatments, such as pentamidine, miltefosine, and amphotericin B, also have notable contraindications and side effects [1].

In addition to systemic therapies, several local treatment modalities have also been evaluated. Cryotherapy with liquid nitrogen is an effective local therapy. It destroys amastigotes and promotes the release of antigenic compounds that enhance the host immune response [5]. The procedure consists of three freeze applications per session, each lasting 15-20 seconds and extending 1-2 mm beyond the lesion margins. Sessions are performed every three weeks [1,3]. Cryotherapy is considered safe during pregnancy and lactation, with minimal side effects [6].

Systematic reviews have shown similar efficacy for cryotherapy and pentavalent antimonials when used as monotherapy, with reported success rates of 67.3% and 67.7%, respectively [1]. One study demonstrated that combined therapy with cryotherapy and intralesional meglumine antimoniate is more effective than monotherapy [7]. Itraconazole has shown efficacy at doses of 200-400 mg daily for one to two months, whereas fluconazole has not proven effective [8,9].

Cryotherapy, oral medications (such as dapsone and itraconazole), and topical imiquimod have been evaluated both as monotherapy and in combination. Monotherapy achieved a success rate of 56.4%, while combination therapy reached 69.5%, with the combination of cryosurgery and itraconazole being the most effective [10].

However, many of these studies have methodological limitations, including small sample sizes, heterogeneity in cure definitions, and regional differences in Leishmania species, making direct comparisons challenging.

Although itraconazole has demonstrated efficacy in the treatment of cutaneous leishmaniasis [11], its use remains less common than that of miltefosine or amphotericin B, which are supported by larger clinical trials and broader international guidelines [4]. Nevertheless, miltefosine is associated with teratogenic risk and frequent gastrointestinal adverse effects, and its cost may limit accessibility in many endemic areas [12]. Amphotericin B, while effective, requires intravenous administration and hospitalization, and is associated with a high risk of nephrotoxicity, particularly with conventional formulations [13]. By contrast, itraconazole is an oral agent with a favorable safety profile and low toxicity, making it a practical and well-tolerated alternative in selected patients. In this case, its use was guided by local availability, safety considerations, and the feasibility of outpatient management as exemplified by the clinical success observed in this report.

## Conclusions

We conclude that the treatment of cutaneous leishmaniasis remains a challenge due to the limited availability of first-line therapies, their adverse effects, high costs, and contraindications. New therapeutic alternatives, such as cryotherapy, may be considered in selected cases. We emphasize the importance of reporting this clinical case, as there are no existing reports of cutaneous leishmaniasis caused by New World *Leishmania* species treated with the combination of cryotherapy and itraconazole.

This case highlights the need for individualized treatment approaches, particularly in regions with restricted access to standard therapies. Practical and well-tolerated alternatives can significantly improve patient adherence and outcomes. Clinical awareness and flexibility in treatment planning are essential, especially when managing parasitic diseases in endemic and resource-limited settings.
